# When do we need clinical endpoint adjudication in clinical trials?

**DOI:** 10.1080/03009734.2018.1516706

**Published:** 2018-11-14

**Authors:** Claes Held

**Affiliations:** Uppsala Clinical Research Center, Department of Medical Sciences, Cardiology, Uppsala, Sweden

**Keywords:** Adjudication, clinical trials, endpoints

## Abstract

Clinical endpoint adjudication (CEA) is a standardized process for assessment of safety and efficacy of pharmacologic or device therapies in clinical trials. CEA plays a key role in many large clinical trials with the aim of achieving consistency and accuracy of the study results, by applying independent and blinded evaluation of suspected clinical events reported by investigators. However, due to high costs there are different opinions regarding the use of central adjudication versus more simplified strategies or site-based assessments and whether the final results differ significantly. There is a lack of scientific evaluation of different adjudication strategies, and more knowledge is needed on the optimal adjudication process and how to achieve the best cost-effectiveness. New methodologies using national registry data and artificial intelligence may challenge the traditional adjudication strategy and could potentially reduce cost considerably with a similar result. Further research and evidence in this field of clinical trials methodology are essential.

## Introduction

Clinical endpoint adjudication (CEA) is a standardized process for the assessment of safety and efficacy of pharmacologic or device therapies in clinical trials. Central adjudication plays a key role in many large clinical trials with the aim of achieving consistent, accurate, independent, unbiased, and blinded evaluation of suspected clinical events reported by investigators. However, there is a controversy regarding the use of central adjudication committees versus simplified strategies or site-based assessments, and, thus, the value has been debated (1,2). Arguments have been made that the final results do not differ from the investigator judgement and that it is a costly process. Despite a long history of centralized adjudication of both cardiovascular (CV) and non-CV endpoint events, there is a need for the clinical trials community to establish a set of best practices to inform how CEA committees should be structured and operated. For a long time there has not been a standardized and commonly accepted CEA process in the scientific community, and there is a need for standardization of the CEA methodology. In addition, historically the endpoint definitions have been inconsistent between studies, which has been a challenge when comparing results between studies. One example is the definition of bleeding, where there are several sets of definitions available. Often, a single study includes several bleeding definitions to adjust for this challenge. However, recently an international expert group, the Bleeding Academic Research Consortium (BARC), developed the BARC standardized bleeding criteria which are now widely accepted (3). Another important endpoint in CV outcome trials is the definition of myocardial infarction (MI), which has been updated several times by the Universal Definitions expert group (4), and a new updated definition is awaited shortly. Recently, an expert committee, led by the Food and Drug Administration (FDA), published a joint suggestion on endpoint definitions of the most important cardiovascular events (5), which is now becoming a standard. For device trials the Academic Research Consortium (ARC) just published suggestions for revised endpoint definitions, such as peri-procedural MIs and stent thrombosis in coronary device trials (6).

Previous studies have shown that the rate of MI assessed by standardized adjudication or by investigator-reported results differed significantly (7). Differential ways of identifying endpoints, such as using triggered events or screening of laboratory data or ECG for MI, could possibly increase the detection of MI (8) or bleedings (9).

At Uppsala Clinical Research Center (UCR), we have offered CEA services for clinical trials for 10 years and have now substantial experience of adjudication of outcomes, from small academic studies to registry-based randomized clinical trials and large big pharma phase III trials for registration purposes. This review aims to provide an overview and highlight the advantages but also the challenges of performing centralized CEA and to provide some future perspectives for the role of central adjudication in future clinical trials.

## Selection of endpoints

When a prospective clinical trial is planned, the primary and secondary outcomes are usually defined as both a composite of clinically relevant outcomes, such as CV death, myocardial infarction (MI), and stroke, and its separate components. In studies on antithrombotic or anticoagulant therapy, safety endpoints are mandatory, of which bleeding complication is an important and common endpoint. When total mortality is included, subcategories such as CV death and non-CV death (infection, cancer, or other organ-specific endpoints) are often being evaluated. Subcategories of CV endpoints, such as non-ST elevation MI versus ST-elevation MI (NSTEMI versus STEMI) or type 1–5 MI, or ischemic versus hemorrhagic stroke, are used for subgroup analyses. Despite the fact that clinical endpoint adjudication has been applied for several decades, the evidence is surprisingly scarce on what endpoint definitions should be used and how to capture the information. Updates and revisions are necessary since the diagnostic tools such as biomarkers and new technology with highly sensitive methods develop with time.

## The CEA process

The CEA process is believed to enhance the validity of clinical trial CV outcome measures through independent, systematic, and standardized identification, processing, and review of CV events. However, little is published that critically and consistently reports on the specifics of CEA organization and the process methodology, and there is no gold standard. The CEA charter is the document that regulates all aspects of the study specifics regarding CEA and to which there should be a reference in the study protocol. Here, the CEA committee is specified, usually composed of clinical specialists in the respective fields, such as cardiology, stroke medicine/neurology, or nephrology. The endpoint office (EPO) collects clinically relevant documents, such as hospital notes, relevant ECGs, lab reports, CT reports, and angiography documentation. The EPO is usually composed of CEA coordinators, monitors, and assistants. The coordinator leads the daily work together with the CEA chair or co-chair.

**Figure 1.**
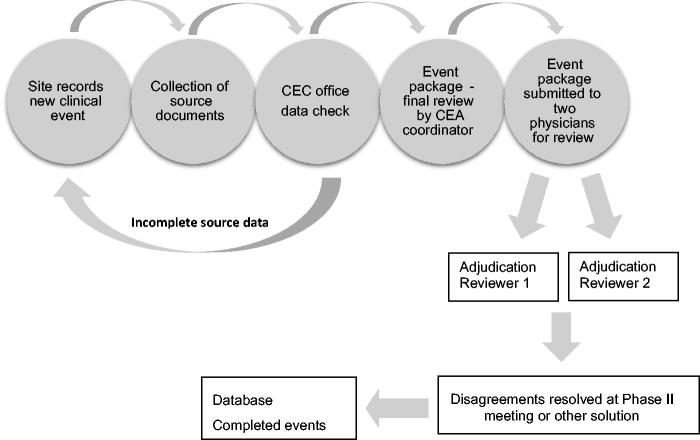
Flow chart of the CEA process.

Collection of predefined source documents has always been challenging in large studies with a high number of endpoints. It used to be paper or fax documents, but with modern technology commercial software is now available that enables the CEA to be carried out completely electronically. The EPO creates PDF files with the relevant endpoint information and submits the package to the reviewers after careful review for completeness (Figure 1). There is no evidence-based scientific evidence on how the flow of events and review process should be handled. However, the most common strategy used in the large global CEA centers is that two independent reviewers evaluate each potential event. If there is agreement on all aspects, the event is complete. In case of disagreement, a process of resolving this is initiated. Depending on the level of disagreement, it may be a committee meeting with several senior specialists coming to a consensus decision or that the two initial reviewers come to a mutual agreement.

Collection of the required source documents should be specified for each type of endpoint. This should be handled by a query process at each site in the study. When this is completed a package of all documents is created as a PDF file. This also often includes some pre-selected relevant data from the eCRF (often called patient profile).

## Registry-based studies

During the last few years, registry-based randomized controlled studies (RRCT) in cardiology have become a new exciting concept in clinical research, using quality improvement registries for prospective randomized trials (10). Such studies are characterized by pragmatism, simple design, and non-complex outcome measures, such as total mortality. Endpoint detection through the use of public registries (as used in RRCTs) can be employed in randomized clinical trials instead of the more traditional approach with active screening and central adjudication (10–12). There are several advantages with endpoint detection through public registries, including a rapid inclusion rate, a more representative all-comer population, as well as substantially lower costs compared to a traditional randomized clinical trial (RCT) (13). Furthermore, official healthcare registries collect a much larger array of possible outcomes. However, registry follow-up is largely based on ICD codes that are dependent on the judgement of the local hospital physician and not on a systematic review of study-specific event definitions. ICD codes are often fairly crude and do not catch granularities around an event, such as sub-categories of a death or an MI. Thus, they may sometimes be inaccurate, and there is also a risk of underreporting of events in registries, such as type 2 MIs or bleedings. A standard RCT may thus offer a higher degree of accuracy regarding a limited number of pre-specified clinical endpoints compared to an RRCT. During the last year two RRCTs were completed in which endpoint adjudication was applied in the ascertainment of endpoints (14,15). The reason was that primary and secondary endpoints, such as bleedings and unplanned revascularization, were slightly more complex and difficult to accurately collect in standard registries. The accuracy of clinical endpoint detection using registries only, as compared to active screening, follow-up, and clinical adjudication (as employed in RCTs), is currently unknown and under investigation (15). Published data from Danish registries comparing hospital medical records with and without central adjudication showed modest agreement on an individual patient basis, but the estimated intervention effects were similar. However, the study addressed the issue of central adjudication or its absence and did not compare active patient follow-up (as in RCTs) compared to using registries only (16).

## Future developments

There is a great need for research in the field of CEA, from evaluation of concordance between investigators and adjudication, endpoint definitions, and the optimal CEA process, to whether other ways of endpoint ascertainment can be applied, such as using ICD codes from registries. We have to compare the quality of data and concordance with the newer technologies. Technical innovations are also needed with better adjudication software, user-friendly both from the perspective of a reviewer and from the administrative side. Better and more detailed reports that can be used for monitoring of the study progress are another important aspect of improvement.

Finally, electronic health records and registries now offer the potential ability to accelerate evidence development, and applying artificial intelligence methodology in the adjudication of clinical endpoints and is a scenario that might become reality in the near future. Hopefully, that possibly could increase the efficacy and considerably reduce the costs.

## Conclusions

Central adjudication of endpoints, with the aim of achieving consistent and accurate evaluation of suspected clinical events reported by investigators, is currently the gold standard in clinical trials. More knowledge is needed on the optimal adjudication process and how to achieve the best cost-effectiveness. New methods using national registry data and artificial intelligence may challenge the traditional adjudication strategy and could potentially reduce cost considerably, with similar results.
